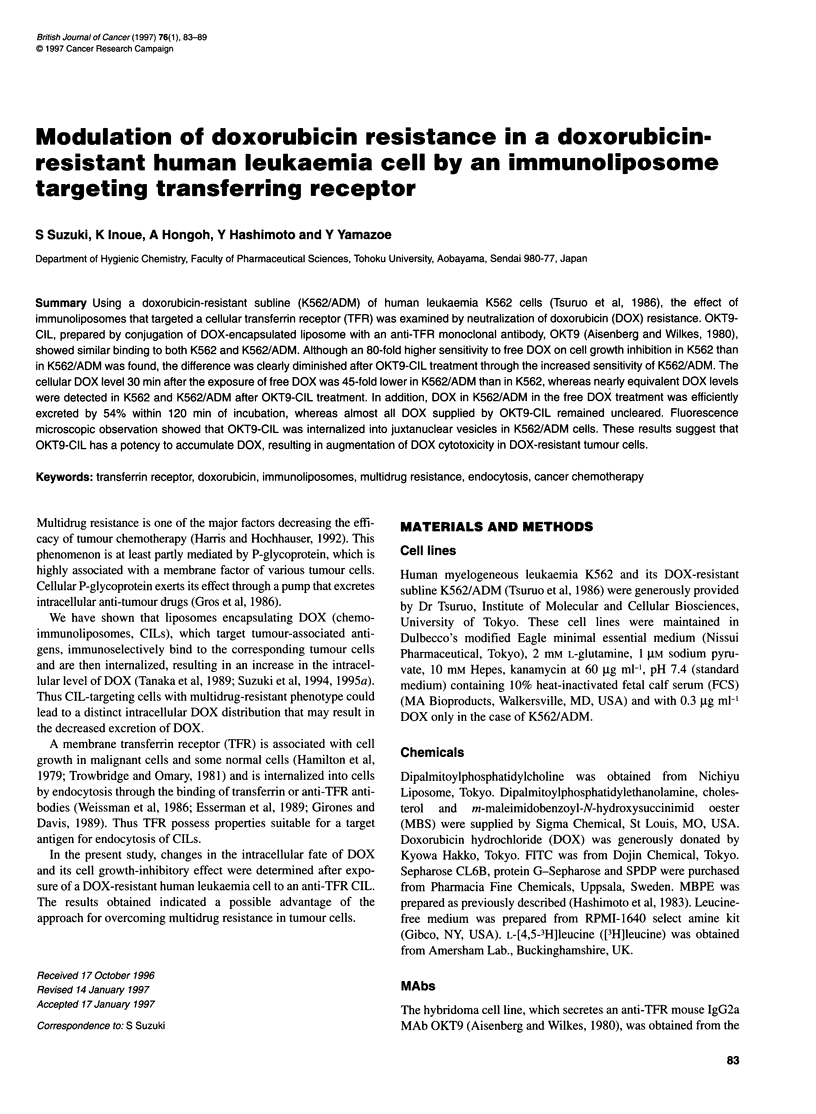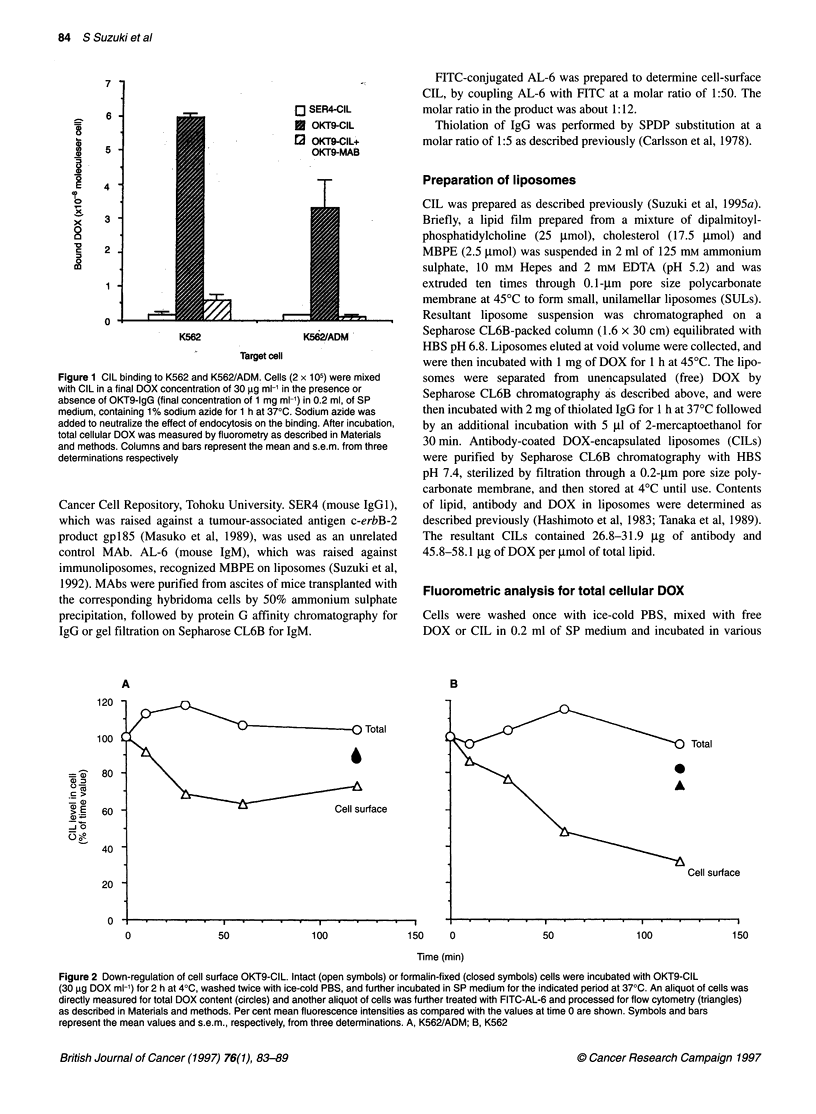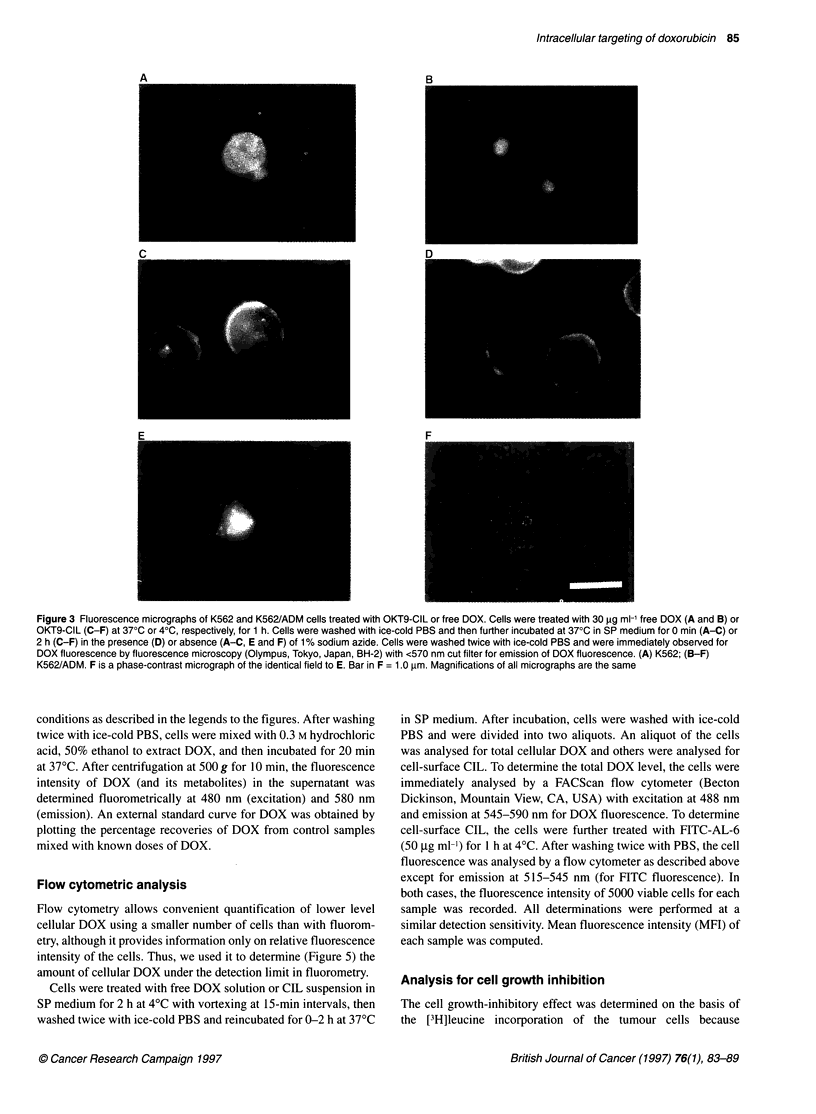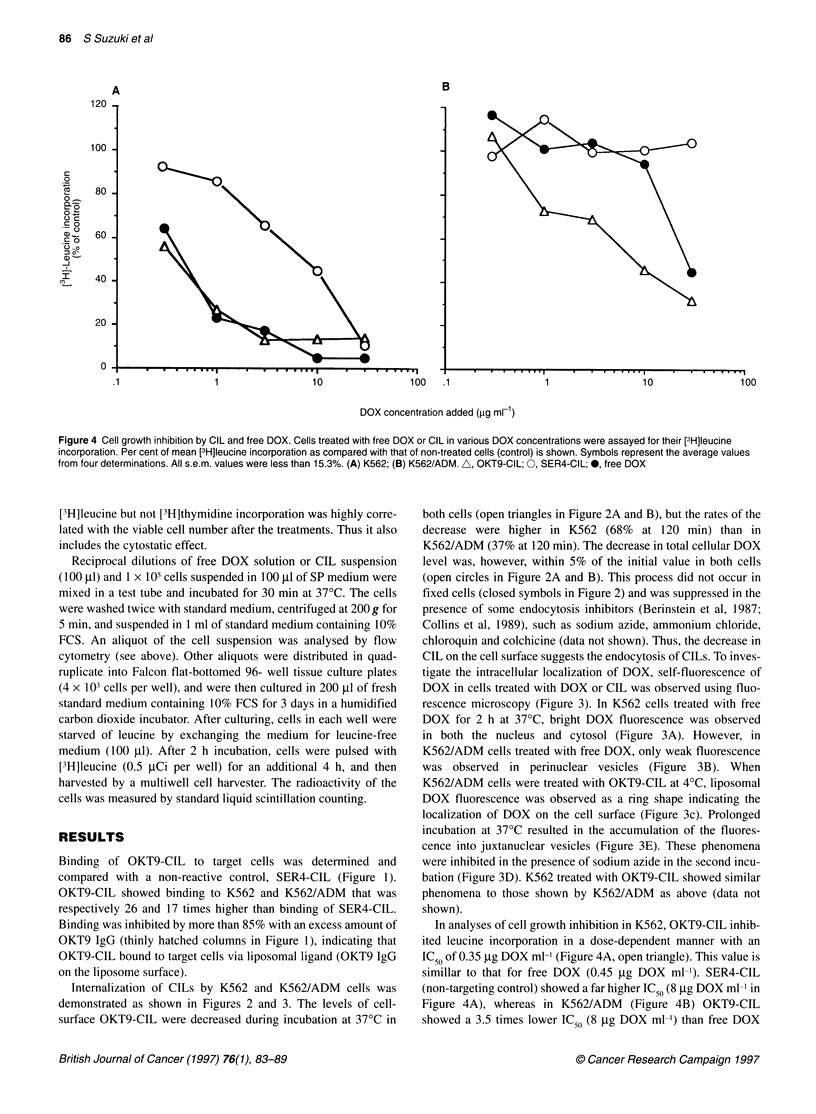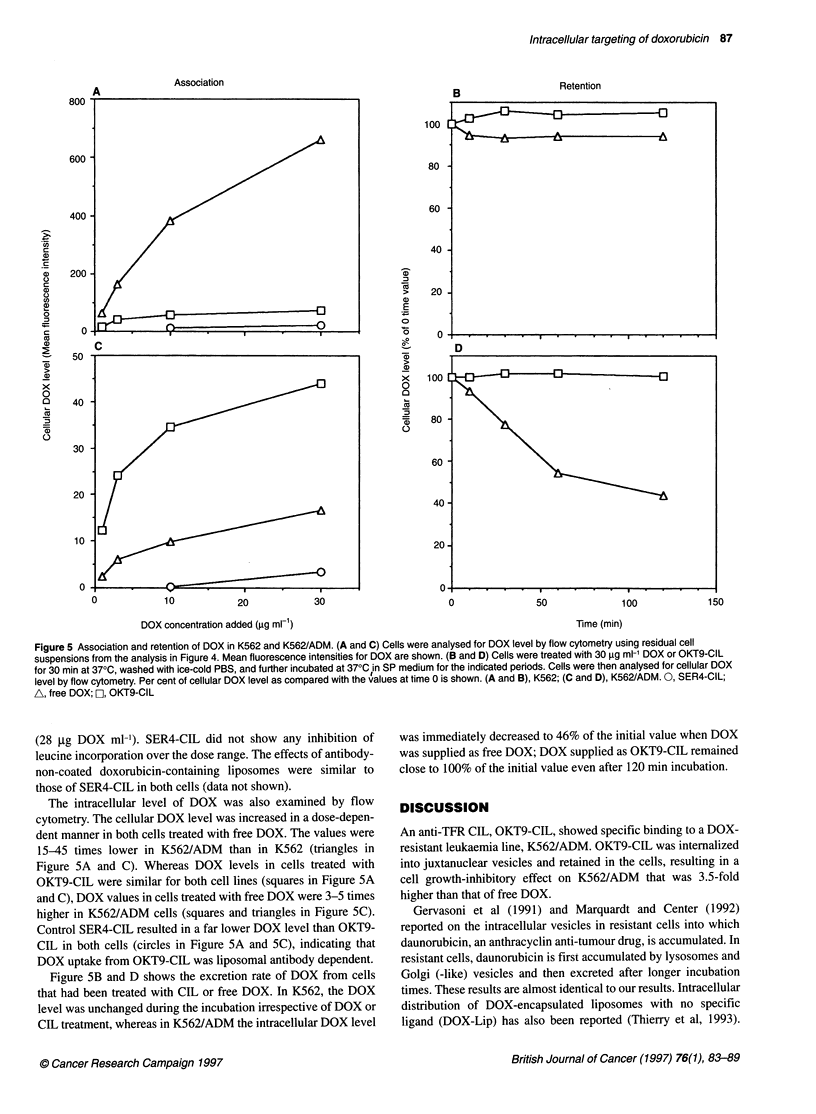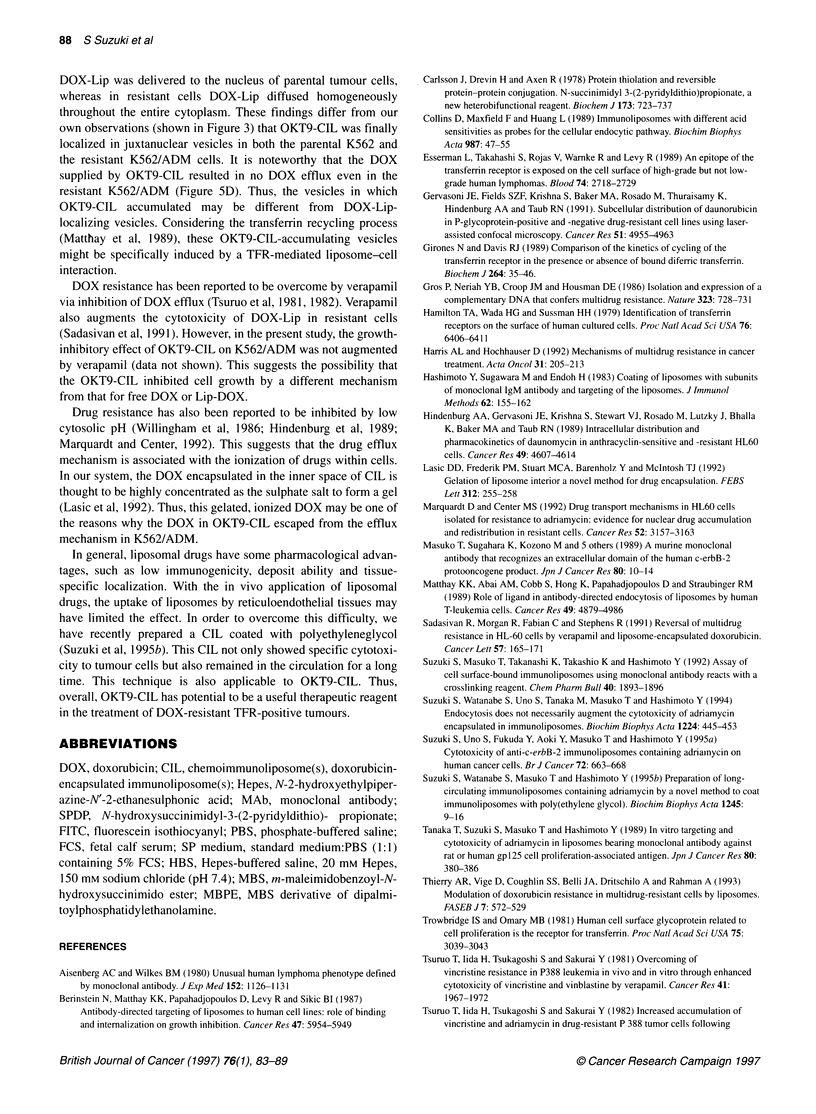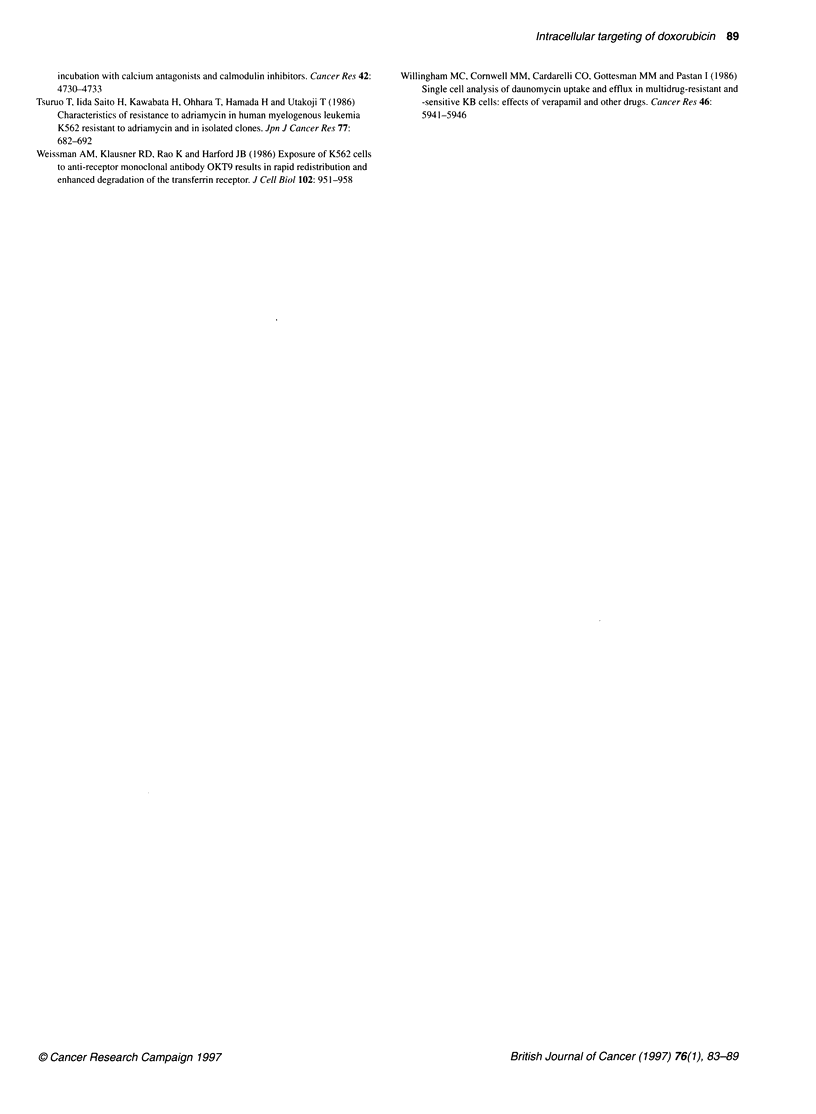# Modulation of doxorubicin resistance in a doxorubicin-resistant human leukaemia cell by an immunoliposome targeting transferring receptor.

**DOI:** 10.1038/bjc.1997.340

**Published:** 1997

**Authors:** S. Suzuki, K. Inoue, A. Hongoh, Y. Hashimoto, Y. Yamazoe

**Affiliations:** Department of Hygienic Chemistry, Faculty of Pharmaceutical Sciences, Tohoku University, Aobayama, Sendai, Japan.

## Abstract

**Images:**


British Journal of Cancer (1997) 76(1), 83–89
© 1997 Cancer Research Campaign

### Modulation of doxorubicin resistance in a doxorubicin-resistant human leukaemia cell by an immunoliposome targeting transferring receptor

**S Suzuki, K Inoue, A Hongoh, Y Hashimoto and Y Yamazoe**

Department of Hygienic Chemistry, Faculty of Pharmaceutical Sciences, Tohoku University, Aobayama, Sendai 980-77, Japan

**Summary** Using a doxorubicin-resistant subline (K562/ADM) of human leukaemia K562 cells (Tsuruo et al, 1986), the effect of immunoliposomes that targeted a cellular transferrin receptor (TFR) was examined by neutralization of doxorubicin (DOX) resistance. OKT9-CIL, prepared by conjugation of DOX-encapsulated liposome with an anti-TFR monoclonal antibody, OKT9 (Aisenberg and Wilkes, 1980), showed similar binding to both K562 and K562/ADM. Although an 80-fold higher sensitivity to free DOX on cell growth inhibition in K562 than in K562/ADM was found, the difference was clearly diminished after OKT9-CIL treatment through the increased sensitivity of K562/ADM. The cellular DOX level 30 min after the exposure of free DOX was 45-fold lower in K562/ADM than in K562, whereas nearly equivalent DOX levels were detected in K562 and K562/ADM after OKT9-CIL treatment. In addition, DOX in K562/ADM in the free DOX treatment was efficiently excreted by 54% within 120 min of incubation, whereas almost all DOX supplied by OKT9-CIL remained uncleared. Fluorescence microscopic observation showed that OKT9-CIL was internalized into juxtanuclear vesicles in K562/ADM cells. These results suggest that OKT9-CIL has a potency to accumulate DOX, resulting in augmentation of DOX cytotoxicity in DOX-resistant tumour cells.

**Keywords:** transferrin receptor, doxorubicin, immunoliposomes, multidrug resistance, endocytosis, cancer chemotherapy

Multidrug resistance is one of the major factors decreasing the efficacy of tumour chemotherapy (Harris and Hochhauser, 1992). This phenomenon is at least partly mediated by P-glycoprotein, which is highly associated with a membrane factor of various tumour cells. Cellular P-glycoprotein exerts its effect through a pump that excretes intracellular anti-tumour drugs (Gros et al, 1986).

We have shown that liposomes encapsulating DOX (chemo-immunoliposomes, CILs), which target tumour-associated antigens, immunoselectively bind to the corresponding tumour cells and are then internalized, resulting in an increase in the intracellular level of DOX (Tanaka et al, 1989; Suzuki et al, 1994, 1995*a*). Thus CIL-targeting cells with multidrug-resistant phenotype could lead to a distinct intracellular DOX distribution that may result in the decreased excretion of DOX.

A membrane transferrin receptor (TFR) is associated with cell growth in malignant cells and some normal cells (Hamilton et al, 1979; Trowbridge and Omary, 1981) and is internalized into cells by endocytosis through the binding of transferrin or anti-TFR antibodies (Weissman et al, 1986; Esserman et al, 1989; Girones and Davis, 1989). Thus TFR possess properties suitable for a target antigen for endocytosis of CILs.

In the present study, changes in the intracellular fate of DOX and its cell growth-inhibitory effect were determined after exposure of a DOX-resistant human leukaemia cell to an anti-TFR CIL. The results obtained indicated a possible advantage of the approach for overcoming multidrug resistance in tumour cells.

*Received 17 October 1996*
*Revised 14 January 1997*
*Accepted 17 January 1997*

*Correspondence to:* S Suzuki

### MATERIALS AND METHODS

### Cell lines

Human myelogeneous leukaemia K562 and its DOX-resistant subline K562/ADM (Tsuruo et al, 1986) were generously provided by Dr Tsuruo, Institute of Molecular and Cellular Biosciences, University of Tokyo. These cell lines were maintained in Dulbecco's modified Eagle minimal essential medium (Nissui Pharmaceutical, Tokyo), 2 mM L-glutamine, 1 μM sodium pyruvate, 10 mM Hepes, kanamycin at 60 μg $ml^{-1}$, pH 7.4 (standard medium) containing 10% heat-inactivated fetal calf serum (FCS) (MA Bioproducts, Walkersville, MD, USA) and with 0.3 μg $ml^{-1}$ DOX only in the case of K562/ADM.

### Chemicals

Dipalmitoylphosphatidylcholine was obtained from Nichiyu Liposome, Tokyo. Dipalmitoylphosphatidylethanolamine, cholesterol and *m*-maleimidobenzoyl-*N*-hydroxysuccinimid oester (MBS) were supplied by Sigma Chemical, St Louis, MO, USA. Doxorubicin hydrochloride (DOX) was generously donated by Kyowa Hakko, Tokyo. FITC was from Dojin Chemical, Tokyo. Sepharose CL6B, protein G–Sepharose and SPDP were purchased from Pharmacia Fine Chemicals, Uppsala, Sweden. MBPE was prepared as previously described (Hashimoto et al, 1983). Leucine-free medium was prepared from RPMI-1640 select amine kit (Gibco, NY, USA). L-[4,5-$^3$H]leucine ([$^3$H]leucine) was obtained from Amersham Lab., Buckinghamshire, UK.

### MAbs

The hybridoma cell line, which secretes an anti-TFR mouse IgG2a MAb OKT9 (Aisenberg and Wilkes, 1980), was obtained from the

**Figure 1** CIL binding to K562 and K562/ADM. Cells ($2 \times 10^5$) were mixed with CIL in a final DOX concentration of 30 μg ml$^{-1}$ in the presence or absence of OKT9-IgG (final concentration of 1 mg ml$^{-1}$) in 0.2 ml, of SP medium, containing 1% sodium azide for 1 h at 37°C. Sodium azide was added to neutralize the effect of endocytosis on the binding. After incubation, total cellular DOX was measured by fluorometry as described in Materials and methods. Columns and bars represent the mean and s.e.m. from three determinations respectively

Cancer Cell Repository, Tohoku University. SER4 (mouse IgG1), which was raised against a tumour-associated antigen c-*erb*B-2 product gp185 (Masuko et al, 1989), was used as an unrelated control MAb. AL-6 (mouse IgM), which was raised against immunoliposomes, recognized MBPE on liposomes (Suzuki et al, 1992). MAbs were purified from ascites of mice transplanted with the corresponding hybridoma cells by 50% ammonium sulphate precipitation, followed by protein G affinity chromatography for IgG or gel filtration on Sepharose CL6B for IgM.

FITC-conjugated AL-6 was prepared to determine cell-surface CIL, by coupling AL-6 with FITC at a molar ratio of 1:50. The molar ratio in the product was about 1:12.

Thiolation of IgG was performed by SPDP substitution at a molar ratio of 1:5 as described previously (Carlsson et al, 1978).

### Preparation of liposomes

CIL was prepared as described previously (Suzuki et al, 1995*a*). Briefly, a lipid film prepared from a mixture of dipalmitoylphosphatidylcholine (25 μmol), cholesterol (17.5 μmol) and MBPE (2.5 μmol) was suspended in 2 ml of 125 mM ammonium sulphate, 10 mM Hepes and 2 mM EDTA (pH 5.2) and was extruded ten times through 0.1-μm pore size polycarbonate membrane at 45°C to form small, unilamellar liposomes (SULs). Resultant liposome suspension was chromatographed on a Sepharose CL6B-packed column ($1.6 \times 30$ cm) equilibrated with HBS pH 6.8. Liposomes eluted at void volume were collected, and were then incubated with 1 mg of DOX for 1 h at 45°C. The liposomes were separated from unencapsulated (free) DOX by Sepharose CL6B chromatography as described above, and were then incubated with 2 mg of thiolated IgG for 1 h at 37°C followed by an additional incubation with 5 μl of 2-mercaptoethanol for 30 min. Antibody-coated DOX-encapsulated liposomes (CILs) were purified by Sepharose CL6B chromatography with HBS pH 7.4, sterilized by filtration through a 0.2-μm pore size polycarbonate membrane, and then stored at 4°C until use. Contents of lipid, antibody and DOX in liposomes were determined as described previously (Hashimoto et al, 1983; Tanaka et al, 1989). The resultant CILs contained 26.8–31.9 μg of antibody and 45.8–58.1 μg of DOX per μmol of total lipid.

### Fluorometric analysis for total cellular DOX

Cells were washed once with ice-cold PBS, mixed with free DOX or CIL in 0.2 ml of SP medium and incubated in various

**Figure 2** Down-regulation of cell surface OKT9-CIL. Intact (open symbols) or formalin-fixed (closed symbols) cells were incubated with OKT9-CIL (30 μg DOX ml$^{-1}$) for 2 h at 4°C, washed twice with ice-cold PBS, and further incubated in SP medium for the indicated period at 37°C. An aliquot of cells was directly measured for total DOX content (circles) and another aliquot of cells was further treated with FITC-AL-6 and processed for flow cytometry (triangles) as described in Materials and methods. Per cent mean fluorescence intensities as compared with the values at time 0 are shown. Symbols and bars represent the mean values and s.e.m., respectively, from three determinations. A, K562/ADM; B, K562

© Cancer Research Campaign 1997

**Figure 3** Fluorescence micrographs of K562 and K562/ADM cells treated with OKT9-CIL or free DOX. Cells were treated with 30 μg ml⁻¹ free DOX (**A** and **B**) or OKT9-CIL (**C–F**) at 37°C or 4°C, respectively, for 1 h. Cells were washed with ice-cold PBS and then further incubated at 37°C in SP medium for 0 min (**A–C**) or 2 h (**C–F**) in the presence (**D**) or absence (**A–C**, **E** and **F**) of 1% sodium azide. Cells were washed twice with ice-cold PBS and were immediately observed for DOX fluorescence by fluorescence microscopy (Olympus, Tokyo, Japan, BH-2) with <570 nm cut filter for emission of DOX fluorescence. (**A**) K562; (**B–F**) K562/ADM. **F** is a phase-contrast micrograph of the identical field to **E**. Bar in **F** = 1.0 μm. Magnifications of all micrographs are the same conditions as described in the legends to the figures. After washing twice with ice-cold PBS, cells were mixed with 0.3 M hydrochloric acid, 50% ethanol to extract DOX, and then incubated for 20 min at 37°C. After centrifugation at 500 ***g*** for 10 min, the fluorescence intensity of DOX (and its metabolites) in the supernatant was determined fluorometrically at 480 nm (excitation) and 580 nm (emission). An external standard curve for DOX was obtained by plotting the percentage recoveries of DOX from control samples mixed with known doses of DOX.

### Flow cytometric analysis

Flow cytometry allows convenient quantification of lower level cellular DOX using a smaller number of cells than with fluorometry, although it provides information only on relative fluorescence intensity of the cells. Thus, we used it to determine (Figure 5) the amount of cellular DOX under the detection limit in fluorometry.

Cells were treated with free DOX solution or CIL suspension in SP medium for 2 h at 4°C with vortexing at 15-min intervals, then washed twice with ice-cold PBS and reincubated for 0–2 h at 37°C in SP medium. After incubation, cells were washed with ice-cold PBS and were divided into two aliquots. An aliquot of the cells was analysed for total cellular DOX and others were analysed for cell-surface CIL. To determine the total DOX level, the cells were immediately analysed by a FACScan flow cytometer (Becton Dickinson, Mountain View, CA, USA) with excitation at 488 nm and emission at 545–590 nm for DOX fluorescence. To determine cell-surface CIL, the cells were further treated with FITC-AL-6 (50 μg ml⁻¹) for 1 h at 4°C. After washing twice with PBS, the cell fluorescence was analysed by a flow cytometer as described above except for emission at 515–545 nm (for FITC fluorescence). In both cases, the fluorescence intensity of 5000 viable cells for each sample was recorded. All determinations were performed at a similar detection sensitivity. Mean fluorescence intensity (MFI) of each sample was computed.

### Analysis for cell growth inhibition

The cell growth-inhibitory effect was determined on the basis of the [$^3$H]leucine incorporation of the tumour cells because

© Cancer Research Campaign 1997

**Figure 4** Cell growth inhibition by CIL and free DOX. Cells treated with free DOX or CIL in various DOX concentrations were assayed for their [$^{3}$H]leucine incorporation. Per cent of mean [$^{3}$H]leucine incorporation as compared with that of non-treated cells (control) is shown. Symbols represent the average values from four determinations. All s.e.m. values were less than 15.3%. (**A**) K562; (**B**) K562/ADM. △, OKT9-CIL; ○, SER4-CIL; ●, free DOX

[$^{3}$H]leucine but not [$^{3}$H]thymidine incorporation was highly correlated with the viable cell number after the treatments. Thus it also includes the cytostatic effect.

Reciprocal dilutions of free DOX solution or CIL suspension (100 µl) and $1 \times 10^5$ cells suspended in 100 µl of SP medium were mixed in a test tube and incubated for 30 min at 37°C. The cells were washed twice with standard medium, centrifuged at 200 ***g*** for 5 min, and suspended in 1 ml of standard medium containing 10% FCS. An aliquot of the cell suspension was analysed by flow cytometry (see above). Other aliquots were distributed in quadruplicate into Falcon flat-bottomed 96- well tissue culture plates ($4 \times 10^3$ cells per well), and were then cultured in 200 µl of fresh standard medium containing 10% FCS for 3 days in a humidified carbon dioxide incubator. After culturing, cells in each well were starved of leucine by exchanging the medium for leucine-free medium (100 µl). After 2 h incubation, cells were pulsed with [$^{3}$H]leucine (0.5 µCi per well) for an additional 4 h, and then harvested by a multiwell cell harvester. The radioactivity of the cells was measured by standard liquid scintillation counting.

### RESULTS

Binding of OKT9-CIL to target cells was determined and compared with a non-reactive control, SER4-CIL (Figure 1). OKT9-CIL showed binding to K562 and K562/ADM that was respectively 26 and 17 times higher than binding of SER4-CIL. Binding was inhibited by more than 85% with an excess amount of OKT9 IgG (thinly hatched columns in Figure 1), indicating that OKT9-CIL bound to target cells via liposomal ligand (OKT9 IgG on the liposome surface).

Internalization of CILs by K562 and K562/ADM cells was demonstrated as shown in Figures 2 and 3. The levels of cell-surface OKT9-CIL were decreased during incubation at 37°C in both cells (open triangles in Figure 2A and B), but the rates of the decrease were higher in K562 (68% at 120 min) than in K562/ADM (37% at 120 min). The decrease in total cellular DOX level was, however, within 5% of the initial value in both cells (open circles in Figure 2A and B). This process did not occur in fixed cells (closed symbols in Figure 2) and was suppressed in the presence of some endocytosis inhibitors (Berinstein et al, 1987; Collins et al, 1989), such as sodium azide, ammonium chloride, chloroquin and colchicine (data not shown). Thus, the decrease in CIL on the cell surface suggests the endocytosis of CILs. To investigate the intracellular localization of DOX, self-fluorescence of DOX in cells treated with DOX or CIL was observed using fluorescence microscopy (Figure 3). In K562 cells treated with free DOX for 2 h at 37°C, bright DOX fluorescence was observed in both the nucleus and cytosol (Figure 3A). However, in K562/ADM cells treated with free DOX, only weak fluorescence was observed in perinuclear vesicles (Figure 3B). When K562/ADM cells were treated with OKT9-CIL at 4°C, liposomal DOX fluorescence was observed as a ring shape indicating the localization of DOX on the cell surface (Figure 3c). Prolonged incubation at 37°C resulted in the accumulation of the fluorescence into juxtanuclear vesicles (Figure 3E). These phenomena were inhibited in the presence of sodium azide in the second incubation (Figure 3D). K562 treated with OKT9-CIL showed similar phenomena to those shown by K562/ADM as above (data not shown).

In analyses of cell growth inhibition in K562, OKT9-CIL inhibited leucine incorporation in a dose-dependent manner with an $IC_{50}$ of 0.35 µg DOX ml$^{-1}$ (Figure 4A, open triangle). This value is simillar to that for free DOX (0.45 µg DOX ml$^{-1}$). SER4-CIL (non-targeting control) showed a far higher $IC_{50}$ (8 µg DOX ml$^{-1}$ in Figure 4A), whereas in K562/ADM (Figure 4B) OKT9-CIL showed a 3.5 times lower $IC_{50}$ (8 µg DOX ml$^{-1}$) than free DOX

 © Cancer Research Campaign 1997

**Figure 5** Association and retention of DOX in K562 and K562/ADM. (**A** and **C**) Cells were analysed for DOX level by flow cytometry using residual cell suspensions from the analysis in Figure 4. Mean fluorescence intensities for DOX are shown. (**B** and **D**) Cells were treated with 30 μg ml⁻¹ DOX or OKT9-CIL for 30 min at 37°C, washed with ice-cold PBS, and further incubated at 37°C in SP medium for the indicated periods. Cells were then analysed for cellular DOX level by flow cytometry. Per cent of cellular DOX level as compared with the values at time 0 is shown. (**A** and **B**), K562; (**C** and **D**), K562/ADM. ○, SER4-CIL; △, free DOX; □, OKT9-CIL

(28 μg DOX ml⁻¹). SER4-CIL did not show any inhibition of leucine incorporation over the dose range. The effects of antibody-non-coated doxorubicin-containing liposomes were similar to those of SER4-CIL in both cells (data not shown).

The intracellular level of DOX was also examined by flow cytometry. The cellular DOX level was increased in a dose-dependent manner in both cells treated with free DOX. The values were 15–45 times lower in K562/ADM than in K562 (triangles in Figure 5A and C). Whereas DOX levels in cells treated with OKT9-CIL were similar for both cell lines (squares in Figure 5A and C), DOX values in cells treated with free DOX were 3–5 times higher in K562/ADM cells (squares and triangles in Figure 5C). Control SER4-CIL resulted in a far lower DOX level than OKT9-CIL in both cells (circles in Figure 5A and 5C), indicating that DOX uptake from OKT9-CIL was liposomal antibody dependent.

Figure 5B and D shows the excretion rate of DOX from cells that had been treated with CIL or free DOX. In K562, the DOX level was unchanged during the incubation irrespective of DOX or CIL treatment, whereas in K562/ADM the intracellular DOX level was immediately decreased to 46% of the initial value when DOX was supplied as free DOX; DOX supplied as OKT9-CIL remained close to 100% of the initial value even after 120 min incubation.

### DISCUSSION

An anti-TFR CIL, OKT9-CIL, showed specific binding to a DOX-resistant leukaemia line, K562/ADM. OKT9-CIL was internalized into juxtanuclear vesicles and retained in the cells, resulting in a cell growth-inhibitory effect on K562/ADM that was 3.5-fold higher than that of free DOX.

Gervasoni et al (1991) and Marquardt and Center (1992) reported on the intracellular vesicles in resistant cells into which daunorubicin, an anthracyclin anti-tumour drug, is accumulated. In resistant cells, daunorubicin is first accumulated by lysosomes and Golgi (-like) vesicles and then excreted after longer incubation times. These results are almost identical to our results. Intracellular distribution of DOX-encapsulated liposomes with no specific ligand (DOX-Lip) has also been reported (Thierry et al, 1993).

© Cancer Research Campaign 1997 

DOX-Lip was delivered to the nucleus of parental tumour cells, whereas in resistant cells DOX-Lip diffused homogeneously throughout the entire cytoplasm. These findings differ from our own observations (shown in Figure 3) that OKT9-CIL was finally localized in juxtanuclear vesicles in both the parental K562 and the resistant K562/ADM cells. It is noteworthy that the DOX supplied by OKT9-CIL resulted in no DOX efflux even in the resistant K562/ADM (Figure 5D). Thus, the vesicles in which OKT9-CIL accumulated may be different from DOX-Lip-localizing vesicles. Considering the transferrin recycling process (Matthay et al, 1989), these OKT9-CIL-accumulating vesicles might be specifically induced by a TFR-mediated liposome–cell interaction.

DOX resistance has been reported to be overcome by verapamil via inhibition of DOX efflux (Tsuruo et al, 1981, 1982). Verapamil also augments the cytotoxicity of DOX-Lip in resistant cells (Sadasivan et al, 1991). However, in the present study, the growth-inhibitory effect of OKT9-CIL on K562/ADM was not augmented by verapamil (data not shown). This suggests the possibility that the OKT9-CIL inhibited cell growth by a different mechanism from that for free DOX or Lip-DOX.

Drug resistance has also been reported to be inhibited by low cytosolic pH (Willingham et al, 1986; Hindenburg et al, 1989; Marquardt and Center, 1992). This suggests that the drug efflux mechanism is associated with the ionization of drugs within cells. In our system, the DOX encapsulated in the inner space of CIL is thought to be highly concentrated as the sulphate salt to form a gel (Lasic et al, 1992). Thus, this gelated, ionized DOX may be one of the reasons why the DOX in OKT9-CIL escaped from the efflux mechanism in K562/ADM.

In general, liposomal drugs have some pharmacological advantages, such as low immunogenicity, deposit ability and tissue-specific localization. With the in vivo application of liposomal drugs, the uptake of liposomes by reticuloendothelial tissues may have limited the effect. In order to overcome this difficulty, we have recently prepared a CIL coated with polyethyleneglycol (Suzuki et al, 1995*b*). This CIL not only showed specific cytotoxicity to tumour cells but also remained in the circulation for a long time. This technique is also applicable to OKT9-CIL. Thus, overall, OKT9-CIL has potential to be a useful therapeutic reagent in the treatment of DOX-resistant TFR-positive tumours.

### ABBREVIATIONS

DOX, doxorubicin; CIL, chemoimmunoliposome(s), doxorubicin-encapsulated immunoliposome(s); Hepes, *N*-2-hydroxyethylpiperazine-*N*′-2-ethanesulphonic acid; MAb, monoclonal antibody; SPDP, *N*-hydroxysuccinimidyl-3-(2-pyridyldithio)- propionate; FITC, fluorescein isothiocyanyl; PBS, phosphate-buffered saline; FCS, fetal calf serum; SP medium, standard medium:PBS (1:1) containing 5% FCS; HBS, Hepes-buffered saline, 20 mM Hepes, 150 mM sodium chloride (pH 7.4); MBS, *m*-maleimidobenzoyl-*N*-hydroxysuccinimido ester; MBPE, MBS derivative of dipalmitoylphosphatidylethanolamine.

### REFERENCES

Aisenberg AC and Wilkes BM (1980) Unusual human lymphoma phenotype defined by monoclonal antibody. *J Exp Med* **152**: 1126–1131

Berinstein N, Matthay KK, Papahadjopoulos D, Levy R and Sikic BI (1987) Antibody-directed targeting of liposomes to human cell lines: role of binding and internalization on growth inhibition. *Cancer Res* **47**: 5954–5949

Carlsson J, Drevin H and Axen R (1978) Protein thiolation and reversible protein–protein conjugation. N-succinimidyl 3-(2-pyridyldithio)propionate, a new heterobifunctional reagent. *Biochem J* **173**: 723–737

Collins D, Maxfield F and Huang L (1989) Immunoliposomes with different acid sensitivities as probes for the cellular endocytic pathway. *Biochim Biophys Acta* **987**: 47–55

Esserman L, Takahashi S, Rojas V, Warnke R and Levy R (1989) An epitope of the transferrin receptor is exposed on the cell surface of high-grade but not low-grade human lymphomas. *Blood* **74**: 2718–2729

Gervasoni JE, Fields SZF, Krishna S, Baker MA, Rosado M, Thuraisamy K, Hindenburg AA and Taub RN (1991). Subcellular distribution of daunorubicin in P-glycoprotein-positive and -negative drug-resistant cell lines using laser-assisted confocal microscopy. *Cancer Res* **51**: 4955–4963

Girones N and Davis RJ (1989) Comparison of the kinetics of cycling of the transferrin receptor in the presence or absence of bound diferric transferrin. *Biochem J* **264**: 35–46.

Gros P, Neriah YB, Croop JM and Housman DE (1986) Isolation and expression of a complementary DNA that confers multidrug resistance. *Nature* **323**: 728–731

Hamilton TA, Wada HG and Sussman HH (1979) Identification of transferrin receptors on the surface of human cultured cells. *Proc Natl Acad Sci USA* **76**: 6406–6411

Harris AL and Hochhauser D (1992) Mechanisms of multidrug resistance in cancer treatment. *Acta Oncol* **31**: 205–213

Hashimoto Y, Sugawara M and Endoh H (1983) Coating of liposomes with subunits of monoclonal IgM antibody and targeting of the liposomes. *J Immunol Methods* **62**: 155–162

Hindenburg AA, Gervasoni JE, Krishna S, Stewart VJ, Rosado M, Lutzky J, Bhalla K, Baker MA and Taub RN (1989) Intracellular distribution and pharmacokinetics of daunomycin in anthracyclin-sensitive and -resistant HL60 cells. *Cancer Res* **49**: 4607–4614

Lasic DD, Frederik PM, Stuart MCA, Barenholz Y and McIntosh TJ (1992) Gelation of liposome interior a novel method for drug encapsulation. *FEBS Lett* **312**: 255–258

Marquardt D and Center MS (1992) Drug transport mechanisms in HL60 cells isolated for resistance to adriamycin: evidence for nuclear drug accumulation and redistribution in resistant cells. *Cancer Res* **52**: 3157–3163

Masuko T, Sugahara K, Kozono M and 5 others (1989) A murine monoclonal antibody that recognizes an extracellular domain of the human c-erbB-2 protooncogene product. *Jpn J Cancer Res* **80**: 10–14

Matthay KK, Abai AM, Cobb S, Hong K, Papahadjopoulos D and Straubinger RM (1989) Role of ligand in antibody-directed endocytosis of liposomes by human T-leukemia cells. *Cancer Res* **49**: 4879–4986

Sadasivan R, Morgan R, Fabian C and Stephens R (1991) Reversal of multidrug resistance in HL-60 cells by verapamil and liposome-encapsulated doxorubicin. *Cancer Lett* **57**: 165–171

Suzuki S, Masuko T, Takanashi K, Takashio K and Hashimoto Y (1992) Assay of cell surface-bound immunoliposomes using monoclonal antibody reacts with a crosslinking reagent. *Chem Pharm Bull* **40**: 1893–1896

Suzuki S, Watanabe S, Uno S, Tanaka M, Masuko T and Hashimoto Y (1994) Endocytosis does not necessarily augment the cytotoxicity of adriamycin encapsulated in immunoliposomes. *Biochim Biophys Acta* **1224**: 445–453

Suzuki S, Uno S, Fukuda Y, Aoki Y, Masuko T and Hashimoto Y (1995*a*) Cytotoxicity of anti-c-*erb*B-2 immunoliposomes containing adriamycin on human cancer cells. *Br J Cancer* **72**: 663–668

Suzuki S, Watanabe S, Masuko T and Hashimoto Y (1995*b*) Preparation of long-circulating immunoliposomes containing adriamycin by a novel method to coat immunoliposomes with poly(ethylene glycol). *Biochim Biophys Acta* **1245**: 9–16

Tanaka T, Suzuki S, Masuko T and Hashimoto Y (1989) In vitro targeting and cytotoxicity of adriamycin in liposomes bearing monoclonal antibody against rat or human gp125 cell proliferation-associated antigen. *Jpn J Cancer Res* **80**: 380–386

Thierry AR, Vige D, Coughlin SS, Belli JA, Dritschilo A and Rahman A (1993) Modulation of doxorubicin resistance in multidrug-resistant cells by liposomes. *FASEB J* **7**: 572–529

Trowbridge IS and Omary MB (1981) Human cell surface glycoprotein related to cell proliferation is the receptor for transferrin. *Proc Natl Acad Sci USA* **75**: 3039–3043

Tsuruo T, Iida H, Tsukagoshi S and Sakurai Y (1981) Overcoming of vincristine resistance in P388 leukemia in vivo and in vitro through enhanced cytotoxicity of vincristine and vinblastine by verapamil. *Cancer Res* **41**: 1967–1972

Tsuruo T, Iida H, Tsukagoshi S and Sakurai Y (1982) Increased accumulation of vincristine and adriamycin in drug-resistant P 388 tumor cells following

© Cancer Research Campaign 1997

incubation with calcium antagonists and calmodulin inhibitors. *Cancer Res* **42**: 4730–4733

Tsuruo T, Iida Saito H, Kawabata H, Ohhara T, Hamada H and Utakoji T (1986) Characteristics of resistance to adriamycin in human myelogenous leukemia K562 resistant to adriamycin and in isolated clones. *Jpn J Cancer Res* **77**: 682–692

Weissman AM, Klausner RD, Rao K and Harford JB (1986) Exposure of K562 cells to anti-receptor monoclonal antibody OKT9 results in rapid redistribution and enhanced degradation of the transferrin receptor. *J Cell Biol* **102**: 951–958

Willingham MC, Cornwell MM, Cardarelli CO, Gottesman MM and Pastan I (1986) Single cell analysis of daunomycin uptake and efflux in multidrug-resistant and -sensitive KB cells: effects of verapamil and other drugs. *Cancer Res* **46**: 5941–5946

© Cancer Research Campaign 1997